# Happiness Through HateLess? Examining the Direct and Indirect Effects of an Anti‐Hate Speech Program on Victimized and Non‐Victimized Youth

**DOI:** 10.1002/jad.12525

**Published:** 2025-06-01

**Authors:** Sebastian Wachs, Catherine Schittenhelm, Maxime Kops, Manuel Gámez‐Guadix, Michelle F. Wright

**Affiliations:** ^1^ Institute of Education, Faculty of Education and Social Science University of Münster Germany; ^2^ Biological and Health Psychology Department Autonomous University of Madrid Madrid Spain; ^3^ Department of Psychology Indiana State University Terre Haute USA

**Keywords:** adolescents, classroom climate, hate speech, prevention, schools, well‐being

## Abstract

**Introduction:**

This study investigated how a 1‐week anti‐hate speech intervention fosters positive classroom dynamics and improves adolescent well‐being. Drawing on Social Capital Theory, we examined whether classroom cohesion enhances happiness among victimized and non‐victimized students.

**Methods:**

A sample of 820 adolescents aged 12 to 16 (*M* = 13.27, SD = 1.04) from 11 German schools was divided into an intervention group (*n* = 567), which participated in the intervention, and a control group (*n* = 253). Self‐report measures of classroom cohesion, happiness, and hate speech victimization were administered before the intervention (T1) and again 1 month later (T2).

**Results:**

Multilevel mediation path analysis revealed that, within the victim subsample, the intervention had a significant direct effect on T2 happiness and mediated effect through classroom cohesion, indicating partial mediation. Conversely, in the non‐victim subsample, the effect of the intervention on T2 happiness was fully mediated by classroom cohesion. Hence, the increase in happiness resulting from the intervention can be fully explained by improvements in classroom cohesion. This suggests that, in contrast to non‐victims, victimized students derive unique direct benefits from the intervention, in addition to the positive impact of enhanced classroom cohesion.

**Conclusions:**

The study underscores the significance of fostering strong social networks in educational settings as a key to student happiness. These insights point toward the potential benefits of tailored intervention approaches, particularly for students facing challenges related to victimization, and highlight the broader educational value of initiatives that build social capital within classrooms.

## 1 Introduction

Hate speech encompasses any form of expression, such as posts, comments, text messages, videos, and images, that aims to humiliate, offend, harm, or exclude individuals based on group characteristics like ethnicity, gender, sexual orientation, or religious affiliation (Kansok‐Dusche et al. 2023). Among youth, hate speech is increasingly prevalent (Castellanos et al. 2023), posing significant threats to both individual well‐being and the overall social climate in schools (Dreißigacker et al. 2024; Krause et al. 2021). Given their pivotal role in shaping students' social interactions and values, schools are uniquely positioned to address and mitigate the adverse effects of hate speech (United Nations 2023). Despite the developmental risks associated with hate speech for youth, there is a notable lack of empirically validated school‐based programs designed to combat hate speech and its far‐reaching consequences (Pfetsch and Ulucinar 2023; Seemann‐Herz et al. 2022).

Theoretical frameworks, such as Social Capital Theory (Bourdieu 1986; Van Rossem et al. 2015), highlight the importance of fostering positive classroom dynamics and building strong interpersonal relationships to enhance students' well‐being. By promoting social capital – through increased trust, cooperation, and mutual respect within the classroom – anti‐hate speech prevention programs may amplify their effectiveness in mitigating the negative effects of hate speech on youth. Building on the Social Capital Theory, this study examined the direct effects of the prevention program HateLess on student happiness and its indirect effects through classroom cohesion. It also explored whether these effects differ between students who have experienced hate speech victimization and those who have not. By investigating these differential impacts, the study contributes to a deeper understanding of how school‐based anti‐hate speech interventions function and for whom they are most effective. Such insights are essential for developing more targeted and responsive programs that support both victimized and non‐victimized students – ultimately fostering well‐being and cohesion across the entire school community.

### 1.1 Description of the HateLess Intervention

HateLess is a science‐based prevention program designed for secondary school students aged 12 to 16 years old. The program's primary objectives are to raise awareness of hate speech, promote prosocial behaviors, and equip students with effective strategies for preventing and addressing incidents of hate speech. Additionally, HateLess aims to foster a culture of respect, inclusion, and unity throughout the entire school community. The main theoretical foundation of HateLess is the socio‐ecological model, which addresses the intrapersonal, interpersonal, and contextual factors that contribute to hate speech (Bauman et al. 2021). This multilevel approach ensures a comprehensive examination of hate speech, considering, for instance, the interplay of personal skills, peer dynamics, and structural influences within the school and the wider social context.

The HateLess curriculum is delivered in five modules, comprising 12 consecutive 90‐min daily sessions each, within a single school week. Recent meta‐analytic work (Kim et al. 2022) indicates that social‐emotional interventions, such as HateLess, are most effective when learners experience multiple sessions (11–20 sessions and sessions over 51 min) that allow for practice and feedback. This high‐engagement format minimizes timetable disruption, providing students with repeated opportunities to apply skills. The first module, *What is Hate Speech?*, defines hate speech, helps students to distinguish it from free speech through critical analysis of intent and harm, and differentiates it from related phenomena, such as bullying and discrimination. The second module, *Why Does Hate Speech Exist?*, investigates the psychological, social, and structural factors that drive hate speech, including stereotypes, group dynamics, and power imbalances. The third module, *Which Consequences Can Hate Speech Have?*, emphasizes the personal and societal consequences of hate speech, including its emotional, social, and political effects on individuals and communities. The module also provides coping strategies to foster resilience. The fourth module, *How Can We Deal with Hate Speech?*, encompasses practical strategies, such as counterspeech, assertive communication, and digital literacy techniques, empowering students to support victims and confront perpetrators. Finally, the fifth module, *How do We Become a HateLess School?*, focuses on integrating the principles and strategies learned in earlier modules into a broader, sustainable school‐wide approach. This module emphasizes the collective responsibility of students and school staff in creating a positive and inclusive school culture. Activities are designed to encourage collaboration, long‐term planning, and the institutionalization of prosocial norms and practices.

The didactical approach of HateLess emphasizes diverse and interactive teaching methods to engage participants effectively. The program integrates experiential learning techniques, such as role‐plays and simulations, allowing participants to practice counterspeech in a safe environment. Group discussions and collaborative activities encourage critical reflection on hate speech and its underlying mechanisms while promoting perspective‐taking and empathy. Media analysis exercises enable students to develop digital literacy skills, such as identifying and countering harmful online content. Additionally, project‐based learning activities provide participants with the opportunity to apply their knowledge and create tangible outputs, such as school‐wide campaigns or digital content promoting inclusivity.

### 1.2 Understanding the Relationship between Classroom Cohesion and Happiness through the Social Capital Theory

Social Capital Theory (Bourdieu 1986) posits that the networks, norms, and trust embedded in interpersonal relationships provide valuable resources that enhance individual well‐being. In schools, the classroom acts as a critical social network where these resources are developed (Van Rossem et al. 2015), shaping students' experiences and interactions. This dynamic is evident in classroom climate, a multifaceted construct that encompasses interpersonal relationships, classroom management, emotional atmosphere, teaching practices, and the physical environment (Loukas 2007). The present study specifically focuses on classroom cohesion, a key component of classroom climate. Classroom cohesion refers to the quality of student relationships and the effectiveness of teamwork within the classroom. It includes solidarity, cooperation, mutual support, and a shared sense of belonging to a unified group with common norms and values (Shapiro 1993). From the perspective of Social Capital Theory, cohesive classroom environments enhance solidarity and teamwork and serve as a source of individual well‐being.

Happiness, as conceptualized in the EPOCH (Engagement, Perseverance, Optimism, Connectedness, and Happiness) model of adolescent well‐being, reflects a stable positive mood characterized by cheerfulness, joy, and a love for life (Kern et al. 2016). In this study, we focus on classroom happiness – a positive emotional state shaped by the quality of social interactions within the classroom (Buerger et al. 2023). Cohesive classrooms foster trust, mutual support, and a shared sense of belonging, which serve as valuable sources of social capital. From this perspective, happiness emerges as both an outcome of strong social bonds and a contributor to broader social and emotional development. Eventually, a cohesive classroom climate equips students with the social and emotional resources needed to navigate challenges, develop interpersonal skills, and build resilience (Kutsyuruba et al. 2015; Lombas et al. 2019). This dual role underscores the importance of classroom cohesion, not only in fostering immediate happiness but also in promoting long‐term flourishing (Kern et al. 2016; Wang et al. 2020).

### 1.3 Differential Effects of HateLess on Classroom Cohesion and Happiness for Hate Speech Victims and Non‐Victims

HateLess actively engages students in developing anti‐hate speech policies and social norms, fostering a sense of ownership and collective responsibility. This collaborative approach strengthens student bonds and enhances unity within the classroom. By collaborating on these initiatives, students develop teamwork and mutual support, which are key components of a cohesive learning environment. HateLess also incorporates practical activities, such as role‐playing, promoting civic courage, and bystander intervention. In this way, the classroom's social fabric is reinforced as students support one another in combating hate speech. Previous research demonstrated the effectiveness of similar strategies in improving the classroom climate (Donohoe 2020; McGiboney 2023). Specifically, evaluation studies have shown that HateLess successfully enhances an inclusive classroom environment and fosters greater cohesion (Wachs et al. 2025). Additionally, the HateLess program has proven effective in developing essential skills like empathy and self‐efficacy, which are vital for nurturing positive and supportive learning environments (Krause and Wachs 2025; Wachs et al. 2023; Wachs et al. 2024a, 2024b). Whether HateLess also impacts adolescents' well‐being (i.e., happiness), particularly regarding distinct effects on victims and non‐victims of hate speech, is not known yet.

The HateLess program encompasses several topics and actions that can directly enhance the happiness of students who have been victimized. For example, its emphasis on practical strategies – such as assertiveness training, counterspeech techniques, and guidance on seeking help – provides victims with the tools they need to navigate and address instances of hate speech. These skills may reduce feelings of helplessness and foster a greater sense of control over their social interactions. This empowerment not only helps victims cope with ongoing or past negative experiences but also builds their resilience, contributing to improved emotional well‐being and confidence in handling challenging situations.

In addition to these direct benefits, the program may also have substantial indirect effects on victims' happiness through its focus on fostering classroom cohesion and increasing counter speech (Wachs et al. 2025; Wachs et al. 2023). By promoting and encouraging peer support, HateLess creates a more supportive and accepting environment within the classroom and serves as a valuable source of social capital. This sense of belonging is particularly beneficial for victimized students, as it counteracts the isolation and exclusion often associated with hate speech experiences. Feeling connected to a cohesive and inclusive group can buffer the negative psychological impacts of victimization (Krause et al. 2021; Wachs et al. 2022) and restore a sense of safety and community. These indirect effects may further enhance the program's overall impact, making it a valuable intervention for addressing the needs of students targeted by hate speech.

Hate speech victimization can have a profound psychological impact and is increasingly recognized as a potentially traumatic experience, especially when it targets core aspects of identity such as ethnicity, religion, or sexual orientation (Allwood et al. 2022; Leets 2002). Victims often report reduced well‐being, heightened distress, and a deep sense of insecurity (Dreißigacker et al. 2024; Wachs et al. 2022; Wypych and Bilewicz 2024). These experiences can elicit trauma‐like responses, including emotional withdrawal, heightened vigilance, and difficulties in trust‐building (Allwood et al. 2022; Leets 2002), all of which may impair students' ability to engage safely and confidently in the classroom (Brunzell et al. 2015). This perspective highlights the importance of designing school‐based interventions that are attuned to both social group dynamics and the individual vulnerabilities of students with a history of victimization.

Although facilitators of HateLess do not receive formal trauma‐informed training, the program manual includes structured guidance for managing emotional distress in the classroom. This includes “pause‐and‐talk” procedures, opportunities for students to temporarily step out of sessions, and referral pathways to school counseling services. These built‐in support mechanisms represent a foundational step toward trauma‐sensitive implementation and help ensure that the program can be delivered in a way that prioritizes students' emotional safety alongside social learning goals.

For non‐victimized students, the program may not have a direct impact on their happiness in the same way. However, they can benefit indirectly through the program's impact on classroom cohesion. By fostering peer support and promoting a respectful and harmonious classroom environment, HateLess contributes to a positive social climate (Wachs et al. 2025). This enhanced cohesion can lead to a greater sense of connection and belonging for all students. It can potentially reduce social tensions and foster a more supportive peer network. In addition to minimizing the likelihood of hate speech (Wachs et al. 2024a, 2024b), such an environment also provides a secure and inclusive space where non‐victimized students can thrive socially and emotionally. Thus, while the direct benefits of HateLess on happiness may be more pronounced for victims of hate speech, the program's indirect effects on happiness via improved classroom cohesion extend to the entire student body, creating a ripple effect that enhances the overall classroom experience.

### 1.4 The Present Study

The study's primary goal was to examine whether the effects of the HateLess program on students' happiness were mediated by classroom cohesion. Particular attention is paid to the unique benefits for those who have experienced hate speech. For hate speech victims, the program offers direct emotional support, empowerment, coping strategies training, and enhanced social resources that may help increase directly victimized students' happiness, whereas, for non‐victims, the positive effects on happiness may be primarily mediated through improved classroom cohesion. By dissecting these differential pathways, the current research aims to provide a nuanced understanding of how anti‐hate speech interventions can be optimized to support all students and especially those at greater risk of emotional harm due to hate speech experiences. We hypothesized that

Hypothesis 1 Among victimized students, the program's effect on T2 happiness will be partially mediated by T2 classroom cohesion.

Hypothesis 2 For non‐victimized students, the program's effect on T2 happiness will be fully mediated by T2 classroom cohesion.

## 2 Method

### 2.1 Participants

The sample for this study consisted of 820 adolescents aged 12 to 16 years (*M* = 13.27, SD = 1.04) from grades 7 to 9 (7th grade: 39.8%; 8th grade: 40.5%; 9th grade: 19.8%) across 37 classes from 11 schools. Of the total sample, 47.3% identified as female, 51% as male, 1.2% as gender diverse, and 0.5% did not specify their gender. Overall, 35.6% had an immigrant background, 63.3% did not, and 1.1% did not provide this information. The intervention group comprised 567 participants (*M*_age_ = 13.24, SD = 1.07; 45.9% female; 35.8% had an immigrant background), while the control group included 253 participants (*M*_age_ = 13.35, SD = 0.97; 50.6% female; 35.2% had an immigrant background).

### 2.2 Measures

#### 2.2.1 Classroom Cohesion

Four items assessed classroom cohesion (e.g., “In my class, we have a strong sense of community”; Bundeszentrale für politische Bildung 2013). Responses could be given on a scale from “strongly disagree” (1) to “strongly agree” (4). The internal consistency of the scale in the current sample was good: Cronbach's alpha _T1_ = 0.81/_T2_ = 0.82, McDonald's Omega _T1_ = 0.80/_T2_ = 0.81. Confirmatory factor analyses (CFAs) confirmed construct validity: CFA _T1_: χ^2^ = 13.71, *df* = 4, *p* = 0.008, CFI = 0.99, TLI = 0.98, RMSEA = 0.05 [0.03, 0.09], SRMR = 0.02. CFA _T2_: χ^2^ = 11.77, *df* = 4, *p* = 0.038, CFI = 0.99, TLI = 0.98, RMSEA = 0.05 [0.01, 0.09], SRMR = 0.02.

#### 2.2.2 Happiness

To measure happiness, we utilized the four‐item subscale for happiness from the EPOCH‐German‐School (“I feel happy in my school”; Buerger et al. 2023), which adapts the corresponding subscale of the general EPOCH instrument (Kern et al. 2016). We adapted the subscale to the classroom rather than the entire school (“I feel happy in my class”). The response scale ranged from “strongly disagree” (1) to “strongly agree” (4). The internal consistency of the scale in the current sample was good: Cronbach's alpha _T1_ = 0.91/_T2_ = 0.93; McDonald's Omega _T1_ = 0.91/_T2_ = 0.92. CFAs confirmed a good fit: CFA _T1_: χ^2^ = 2.69, *df* = 2, *p* = 0.260, CFI = 0.99, TLI = 0.95, RMSEA = 0.07 [0.05, 0.10], SRMR = 0.03. CFA _T2_: χ^2^ = 2.76, *df* = 2, *p* < 0.251, CFI = 0.99, TLI = 0.99, RMSEA = 0.03 [0.01, 0.09], SRMR = 0.01.

#### 2.2.3 Hate Speech Victimization

After being presented a definition of hate speech, participants were asked to respond to the statement: “In my class, I become the target of hate speech,” using a five‐point scale ranging from 1 (never) to 5 (very often). For comparative analyses, responses were recoded into two categories: a response of 1 (never) was classified as “Non‐victim” (1), while all responses above 1 were classified as “Victim” (2).

#### 2.2.4 Control Variables

Participants were asked to provide their age and gender, with gender options including male, female, and gender diverse. To determine their immigrant background, participants reported their own birthplace and their parents' birthplaces, specifically whether they were born in a country other than Germany. Based on the official German criteria for immigrant background (The Federal Statistical Office, 2022), participants were considered to have an immigrant background if they or at least one of their parents was born outside of Germany.

### 2.3 Procedure

Following approval by the data protection officer and the educational authority of the Federal State of Berlin and the Federal State of Brandenburg (Germany), we approached 34 schools to participate in the study, of which 11 (32% acceptance rate) consented. Across these schools, 44 classes were invited, with seven opting out (84% acceptance rate), leaving 37 classes in total. In these classes, 909 participants were invited; 31 declined, yielding a 96% participation rate (*n* = 878). Of these, 820 participants completed both the pretest (T1) and posttest (T2), with 58 not participating in the posttest. Data were collected from June to November 2022 during regular class sessions, 1 week before the intervention (T1) and 1 month afterward (T2), through computer‐assisted personal interviews administered by trained research assistants.

### 2.4 Data Analysis

We began our analyses by exploring the data with descriptive statistics and independent *t*‐tests to assess the study variables and examine means for baseline (T1) and follow‐up (T2) and bivariate correlations among the main variables in the total sample. Next, we conducted a two‐level multilevel mediation analysis in M*plus* 8.11 (Muthén and Muthén 2024) to evaluate the impact of the HateLess program on classroom cohesion and happiness. First, we computed intraclass correlation coefficients (ICCs) using a random‐intercept‐only model to assess the extent of classroom‐level clustering. We then specified a mediation model to test whether intervention group (vs. control) status predicted T2 classroom cohesion and T2 happiness and whether classroom cohesion mediated the link between intervention status and T2 happiness. Baseline levels of classroom cohesion, baseline happiness, and demographic factors (age, gender, immigrant background) were included as predictors of the T2 outcomes. All predictors were measured at the student level (Level 1), with no classroom‐level (Level 2) predictors included in the model. This mediation analysis was run for three groups: (1) the total sample (Model_T_), (2) adolescents reporting hate speech victimization at T1 (Model_V_), and (3) adolescents without victimization experiences at T1 (Model_NV_). The findings for Model_T_ are reported in the supporting material.

Before comparing results between the victimized and non‐victimized groups, we assessed measurement invariance for classroom cohesion and happiness. We conducted multi‐group CFAs in Mplus using the MLR estimator, following Chen (2007) guidelines. A decrease in ∆CFI exceeding 0.010 and an increase in ∆RMSEA exceeding 0.015 signify a lack of invariance. Metric invariance suffices for comparing structural relationships, such as mediation paths (Steenkamp and Baumgartner 1998). We addressed missing data using the FIML estimator.

Finally, the effect size of the intervention's effect on happiness was calculated using Cohen's *d*, which measures the magnitude of the intervention's impact in standard deviation units. Cohen's *d* was derived from the unstandardized regression coefficients (*B*) provided by the multilevel mediation model in the study. The pooled standard deviation (SD) for each outcome was calculated based on the standard deviations of the intervention and control groups. The formula used to calculate the pooled standard deviation (SD) was as follows:

$$SD_{\mathrm{pooled}}=\sqrt{\frac{\left(n_{1}-1\right)*{\mathrm{SD}}_{1}^{2}+\left(n_{2}-1\right)*{\mathrm{SD}}_{2}^{2}}{n_{1}{+n}_{2}-2}}$$

where *n*_1_ and *n*_2_ are the sample sizes for the intervention and control groups, respectively, and SD_1_ and SD_2_ are the standard deviations for the intervention and control groups.

Once the pooled SD was obtained, Cohen's *d* was calculated using the following formula:

$$d=\frac{B}{SD_{\mathrm{pooled}}}$$

where *B* is the unstandardized regression coefficient from the multilevel mediation model, and SD_pooled_ is the pooled standard deviation calculated in the previous step.

## 3 Results

### 3.1 Baseline Equivalence

The intervention and control group presented a similar distribution regarding age, *t*(814) = 1.12, *p* = 0.130, gender, *X* ^2^ (1, *N* = 806) = 1.36, *p* = 0.244, and immigrant background, *X* ^2^ (1, *N* = 817) = 2.36, *p* = 0.125. Then, *t*‐tests were conducted separately for each baseline variable at the pretest. Results showed no significant differences between the intervention and control groups for T1 classroom cohesion (*t*(813) = 0.111, *p* = 0.456) and T1 hate speech victimization (*t*(811) = 1.44, *p* = 0.075). However, participants reported slightly higher T1 happiness in the control group than in the intervention group, *t*(812) = 2.68, *p* = 0.009, Cohen's *d* = 0.20, 95%CI [0.05; 0.35].

### 3.2 Preliminary Analyses

Table 1 shows the correlations and descriptives of the study's main variables separated by victim and non‐victim subsample. Supplementary Table S1 provides correlations and descriptive statistics for the total sample. Table 2 reports the results of the measurement invariance testing. For T2 classroom cohesion, the changes in fit indices from configural to metric invariance remained below conventional cutoffs (ΔCFI ≤ 0.010 and ΔRMSEA ≤ 0.015), indicating equivalent factor structure and factor loadings across groups (victims vs. non‐victims). However, moving to scalar invariance exceeded these cutoffs, suggesting that item intercepts differ between groups. Consequently, configural and metric invariance are supported, but full scalar invariance is not. For T2 happiness, results showed that the changes in fit indices are below the conventional cutoffs (ΔCFI ≤ 0.010 and ΔRMSEA ≤ 0.015), indicating configural, metric, and scalar invariance. In conclusion, the findings indicate at least metric invariance across the groups for both constructs. Consequently, we could validly compare structural relations (e.g., mediation paths) across groups.

**Table 1:** Correlations and Descriptives among the Study's Main Variables Split by Victims and Non‐victims.

Variable	1	2	3	4
1. T1 Classroom cohesion	—	0.51**	0.48**	0.40**
2. T2 Classroom cohesion	0.31**	—	0.26**	0.50**
3. T1 Happiness	0.47**	0.16*	—	0.47**
4. T2 Happiness	0.25**	0.43**	0.39**	—
*M* (SD) _Victim (total)_	2.94 (0.74)	3.14 (0.66)	2.80 (0.81)	2.94 (0.78)
*M* (SD) _Victim (intervention)_	2.93 (0.75)	3.26 (0.58)	2.77 (0.80)	3.04 (0.69)
*M* (SD) _Victim (control)_	2.87 (0.72)	2.84 (0.77)	2.87 (0.72)	2.75 (0.93)
*M* (SD) _Non‐victim (total)_	3.05 (0.82)	3.30 (0.69)	3.14 (0.80)	3.36 (0.76)
*M* (SD) _Non‐victim (intervention)_	3.01 (0.82)	3.38 (0.74)	3.07 (0.79)	3.39 (0.75)
*M* (SD) _Non‐victim (control)_	3.05 (0.85)	3.07 (0.82)	3.27 (0.83)	3.30 (0.82)

*Note:* **p < 0.001 *p < 0.01. T1 = Pretest, T2 = Posttest. Correlations above the diagonal represent the victim subsample, and correlations below the diagonal represent the non‐victim subsample.

**Table 2:** *Measurement Invariance Testing across Victimization Status*.

Models	χ^2^ (*df*)	*p*	RMSEA	∆ RMSEA	CFI	∆ CFI	Invariance rule accepted
**Model 1: T2 Classroom cohesion**							
Configural	8.86 (4)	0.065	0.055		0.994		
Metric	10.32 (7)	0.171	0.035	−0.020	0.996	0.002	Yes
Scalar	27.14 (10)	0.003	0.066	0.031	0.980	−0.016	No
**Model 2: T2 Happiness**							
Configural	13.74 (4)	<0.001	0.078		0.989		
Metric	24.93 (7)	<0.001	0.080	−0.002	0.982	−0.007	Yes
Scalar	31.76 (10)	<0.001	0.077	−0.003	0.975	−0.007	Yes

### 3.3 Multilevel Mediation Model

Within the victim subsample, the model explained 22% of the variance in T2 classroom cohesion (*R²* = 0.22) and 34% in T2 happiness (*R²* = 0.34). The intervention had a positive effect on T2 classroom cohesion (*B* = 0.47, *SE* = 0.15, *p* = 0.002, *d* = 0.73). Moreover, the direct effect of the intervention on T2 happiness was significant (*B* = 0.32, *SE* = 0.16, *p* = 0.043, *d* = 0.42). T2 classroom cohesion was also positively associated with T2 happiness (*B* = 0.38, *SE* = 0.07, *p* < 0.001). The indirect effect of the intervention on T2 happiness through T2 classroom cohesion was significant, indicating partial mediation (*B* = 0.18, *SE* = 0.07, 95% CI [0.04, 0.32]): Victimized students' happiness increased partly through enhanced classroom cohesion and partly through a direct effect of the intervention. Regarding covariates, age was negatively related to T2 happiness but did not predict T2 classroom cohesion. Gender was unrelated to T2 classroom cohesion and T2 happiness. Finally, having an immigrant background was unrelated to T2 happiness but positively predicted T2 classroom cohesion (see Table 3, Model_V_).

**Table 3:** *Results of the Multilevel Mediation Model Split by Victims and Non‐victims*.

Predictor	Mediator	Outcome	*B* (*SE*)	*p*
**Model**_**V**_				
**Direct Effects**				
Group assignment ^intervention^	Classroom cohesion (T2)		0.47 (0.15)	0.002
Group assignment ^intervention^		Happiness (T2)	0.32 (0.16)	0.043
	Classroom cohesion (T2)	Happiness (T2)	0.38 (0.07)	<0.001
**Indirect Effects**				
Group assignment intervention	Classroom cohesion (T2)	Happiness (T2)	0.18 (0.07) 95% CI [0.04, 0.32]	
**Control variables**				
Age		Classroom cohesion (T2)	−0.04 (0.06)	0.546
Age		Happiness (T2)	0.16 (0.08)	0.041
Gender ^girls^		Classroom cohesion (T2)	0.11 (0.10)	0.290
Gender ^girls^		Happiness (T2)	0.22 (0.14)	0.124
Immigrant background ^yes^		Classroom cohesion (T2)	0.71 (0.31)	0.021
Immigrant background ^yes^		Happiness (T2)	0.15 (0.22)	0.488
Classroom cohesion (T1)		Classroom cohesion (T2)	0.31 (0.11)	0.004
Happiness (T1)		Happiness (T2)	0.33 (0.08)	<0.001
**Model**_**NV**_				
**Direct Effects**				
Group assignment intervention	Classroom cohesion (T2)		0.45 (0.10)	<0.001
Group assignment intervention		Happiness (T2)	−0.01 (0.09)	0.941
	Classroom cohesion (T2)	Happiness (T2)	0.41 (0.05)	<0.001
**Indirect Effects**				
Group assignment intervention	Classroom cohesion (T2)	Happiness (T2)	0.18 (0.05) 95% CI [0.09, 0.28]	
**Control variables**				
Age		Classroom cohesion (T2)	−0.05 (0.10)	0.331
Age		Happiness (T2)	−0.09 (0.03)	0.007
Gender^girls^		Classroom cohesion (T2)	0.18 (0.09)	0.041
Gender^girls^		Happiness (T2)	0.01 (0.07)	0.979
Immigrant background ^yes^		Classroom cohesion (T2)	0.28 (0.11)	0.012
Immigrant background ^yes^		Happiness (T2)	−0.21 (0.14)	0.136
Classroom cohesion (T1)		Classroom cohesion (T2)	0.50 (0.06)	<0.001
Happiness (T1)		Happiness (T2)	0.36 (0.07)	<0.001

*Note:* T1 = Pretest, T2 = Posttest. Reference category: group assignment = control group; gender = boys; immigrant background = no. *V* denotes subsample victims, and *NV* denotes subsample non‐victims.

**Figure 1:**
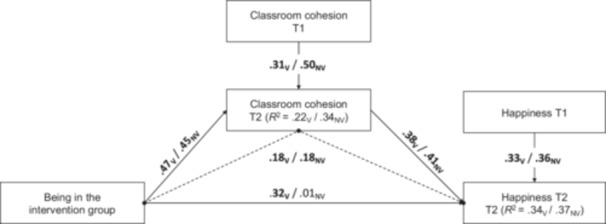
*Direct and Indirect Effects of the Intervention on Happiness via Classroom Cohesion for the Victims and Non‐victims*. *Note:* All effects are unstandardized. Bold coefficients are significant, and regular coefficients are not. *V* denotes subsample victims, and *NV* denotes subsample non‐victims. The dashed line represents the path of the indirect effect. Age, sex, and immigrant background are regressed on all T2 variables but are not shown for ease of interpretation.

Among non‐victimized students, the model explained 34% of the variance in T2 classroom cohesion (*R²* = 0.34) and 37% for T2 happiness (*R²* = 0.37). The intervention continued to predict T2 classroom cohesion (*B* = 0.45, *SE* = 0.10, *p* < 0.001, *d* = 0.64). In contrast to the victim subsample, the direct path from the intervention to T2 happiness was not significant (*B* = −.01, *SE* = 0.09, *p* = 0.941). As in the victim subsample, higher T2 classroom cohesion was positively related to higher T2 happiness *B* = 0.41, *SE* = 0.05, *p* < 0.001). The indirect effect of the intervention's influence on happiness via classroom cohesion was significant, indicating full mediation (*B* = 0.18, *SE* = 0.05, 95% CI [0.09, 0.28]). The finding suggests that in the non‐victim group, the intervention effect on happiness operates exclusively via improvements in classroom cohesion. Regarding covariates, age was negatively related to T2 happiness but did not predict T2 classroom cohesion. Girls reported higher T2 classroom cohesion than boys, though gender was not associated with T2 happiness. Lastly, having a migration background was unrelated to T2 happiness but positively predicted T2 classroom cohesion (see Table 3, Model_NV_). Figure 1 shows the direct and indirect effects of both models. Supplementary Table S2 presents the multilevel mediation model for the total sample (Model_T_).

## 4 Discussion

This study aimed to evaluate whether the HateLess program's effects on students' happiness were mediated by classroom cohesion, focusing on comparing victimized and non‐victimized students. The results highlight the pivotal role of classroom cohesion in mediating the program's effect on students' happiness, with distinct dynamics observed between students who have been victimized and those who have not.

Across both groups and in line with previous research (Kutsyuruba et al. 2015; Lombas et al. 2019; Wang et al. 2020), classroom cohesion was positively associated with happiness. According to the Social Capital Theory (Bourdieu 1986; Van Rossem et al. 2015), a cohesive classroom environment embodies social capital, which offers resources that buffer against stress, enhance resilience, and promote overall well‐being. The intervention had a medium to large short‐term effect on classroom cohesion in both subsamples, which aligns with the broader literature indicating significant positive effects of structured interventions on school climate (Charlton et al. 2021).

For victimized students, the intervention directly and indirectly influenced happiness through classroom cohesion, supporting partial mediation (H1). The intervention had a small to medium short‐term effect on happiness, which aligns with a current meta‐analysis on the effects of school‐based multicomponent interventions aimed at increasing youth's well‐being (Tejada‐Gallardo et al. 2020). Although the intervention yielded statistically significant improvements, it may not fully meet the needs of all students who have experienced chronic or severe hate speech victimization. For these students, additional psychosocial support services – such as counseling, trauma‐informed individual interventions, or school‐based mental health care – may be necessary to complement the effects of universal programs like HateLess.

Incorporating principles from trauma‐informed education frameworks may further enhance the program's effectiveness for students who have been victimized. Trauma‐informed approaches emphasize safety, empowerment, trust‐building, and peer connection (Brunzell et al. 2015) – all elements reflected in the HateLess program's design. Hate speech victimization can be experienced as a form of interpersonal trauma, which can disrupt students' sense of belonging and psychological safety in the classroom (Allwood et al. 2022). Interventions grounded in trauma‐informed practices aim to restore these foundational needs. For example, the HateLess program's emphasis on counterspeech strategies, empathy‐building, and collaborative classroom norms (Wachs et al. 2023; Wachs et al. 2025) aligns with trauma‐informed principles by fostering predictability, agency, and relational security. These elements are especially vital for students with prior exposure to hate speech, as they may be more sensitive to perceived threats or exclusion. Thus, future adaptations of HateLess could further benefit from explicitly embedding trauma‐informed practices, such as staff training in recognizing trauma responses or offering opt‐in modules for students with elevated distress levels.

As an indicator of social capital, classroom cohesion provided emotional support, a sense of belonging, and access to shared resources. These elements are particularly critical for victimized students, who often experience low well‐being (Dreißigacker et al. 2024; Wachs et al. 2022; Wypych and Bilewicz 2024). This finding also aligns with trauma theory: positive peer networks buffer distress and foster empowerment after hate exposure (Allwood et al. 2022; Brunzell et al. 2015). Classroom climate is also positively linked to youth empathy for victims of hate speech and their self‐efficacy in intervening against hate speech (Wachs et al. 2025), both of which may enhance the victim's well‐being. Additionally, previous research showed that in classrooms with a positive climate, students tend to be more likely to defend victims and are less likely to contribute to hate speech. Consequently, hate speech occurs less frequently (Wachs et al. 2024a, 2024b), which might also positively affect victims' well‐being within the classroom.

While enhanced classroom cohesion significantly contributed to increased happiness, the direct impact of the intervention on happiness suggests that victimized students also benefit from additional psychosocial support embedded in the program. This direct effect may stem from targeted elements of the intervention that address their specific vulnerabilities, such as activities fostering emotional resilience or empowering students to navigate adverse experiences. This dual pathway highlights the program's effectiveness in addressing contextual (i.e., classroom‐level) and individual needs.

In contrast, the HateLess's effect on happiness was fully mediated by classroom cohesion for non‐victimized students, supporting a full mediation model (H2). Thus, the intervention primarily functions in this group by cultivating an environment where students feel connected and supported. This finding demonstrates that the program's benefits for subjective well‐being depend largely on the quality of peer relationships and collective classroom dynamics. The results indicate that the program strengthens the social fabric of the classroom and thus aligns with Social Capital Theory (Bourdieu 1986; Van Rossem et al. 2015). A cohesive classroom offers students greater emotional security, enhances self‐esteem, and fosters a shared sense of purpose, and, hence, improves psychological well‐being (Wang et al. 2020). In summary, comparing the findings between victimized and non‐victimized students reveals a structural difference in how the intervention affects the two groups. This underscores the role of personal experience with hate speech: Victimized students may benefit not only from a more cohesive climate but also from the specific coping strategies and empowerment components embedded in the program.

While the HateLess program demonstrated strengths in promoting classroom cohesion and student well‐being, its contribution also becomes clearer when viewed in contrast to other anti‐hate speech programs. The comprehensive analysis by Fischer et al. (2025) identified 27 anti‐hate speech programs used in German schools, revealing considerable variation in theoretical foundation, duration, flexibility, and evaluation practices. Notably, most programs focused on awareness‐raising and counter‐speech strategies but lacked consistent theoretical grounding or summative evaluation designs. In contrast, HateLess is among the few programs with a clear theoretical framework and a focus on outcomes like classroom cohesion and students' well‐being. Moreover, unlike many programs that either adopt a universal or a case‐specific approach, HateLess integrates both preventive and responsive components and considers individual experiences of victimization. This dual focus distinguishes it from many evaluated approaches in the field.

### 4.1 Limitations and Future Research

Despite promising findings, several limitations warrant consideration. First, the study relied on self‐report measures, raising the possibility of social desirability bias and common method variance. Although confidentiality was assured, future research could benefit from multi‐informant (e.g., teacher or peer reports) or observational data to enhance validity. Second, hate speech victimization was measured with a single‐item indicator, potentially underrepresenting the complexity and severity of these experiences. More comprehensive scales or qualitative assessments may capture a broader range of victimization experiences, thereby improving measurement precision. In addition, both classroom climate and well‐being are multidimensional constructs. However, we investigated only one facet of each construct in the present study. Follow‐up research should expand the present findings by considering further dimensions of classroom climate (e.g., teacher‐student relationships) and well‐being (e.g., optimism, engagement).

Third, the relatively short 1‐month follow‐up limits conclusions about the program's long‐term effectiveness. Extended follow‐ups and longitudinal designs are needed to assess the durability of intervention effects over time. Fourth, it remains unclear which specific program components most effectively drive the observed changes. Future research could employ dismantling studies or moderation analyses to identify the intervention's most impactful elements and tailor them to students' specific risk profiles. Fourth, although no formal fidelity logs were collected, a fully scripted manual and immediate access to research team support were offered to maintain uniformity in delivery. Future research should include systematic observation or logging to document adherence in a quantitative and objective manner.

Fifth, a further limitation concerns the lack of data on how facilitators responded to emotional disclosures or student distress during or after the intervention. While the HateLess program was designed with sensitivity to student experiences, we did not systematically assess how facilitators handled emotionally charged moments – an important consideration given the psychological impact hate speech can have, especially for previously victimized students. Trauma‐informed educational frameworks emphasize the importance of emotional regulation and psychological safety in these settings (Brunzell et al. 2015). Future research should therefore examine implementation practices in greater detail, including how facilitators are trained to manage distress, establish safe learning environments, and respond appropriately to students' emotional reactions. Finally, exploring additional mediators may provide a more nuanced understanding of how anti‐hate speech programs exert their effects and further understanding of the intervention's effectiveness for participants with and without a history of victimization. Along the same line, the differential effects of the intervention on hate speech perpetrators and non‐perpetrators should be investigated in follow‐up research.

### 4.2 Practical Implications

The findings of this study carry several important implications for educational practice and policy. First, the findings underscore the importance of cultivating a strong, interconnected classroom community to promote students' overall well‐being and happiness. Educational interventions that emphasize the development of trust, mutual support, and positive peer relationships can substantially bolster the social resources available to students. This, in turn, is positively related to a happier and more resilient student body. Second, for students who have experienced hate speech, the intervention produced both direct and indirect effects on happiness. Hence, tailored approaches that recognize the distinct needs of students who have been victimized are crucial. Ensuring that resources are available to address the emotional harm caused by hate speech and educating students about coping strategies can enhance the specific impact of anti‐hate speech programs for victims. Besides focusing on the special needs of victims to increase their happiness, positive effects for victims are also achieved by the gradual accumulation of social capital in the classroom environment. Therefore, the most promising approach is a dual strategy emphasizing fostering interpersonal connections while directly supporting victims.

### 4.3 Conclusion

This study highlights the transformative potential of anti‐hate speech interventions in educational settings. The HateLess program demonstrated a dual pathway of impact: for victimized students, it provided both direct emotional support and indirect benefits through improved classroom cohesion, while for non‐victimized students, the effects on happiness were fully mediated by the strengthened social fabric of the classroom. These findings underscore the critical role of fostering cohesive and supportive classroom environments in promoting student well‐being and social capital. The results emphasize the practical value of tailored interventions that address both individual vulnerabilities and collective classroom dynamics.

Future research should explore the long‐term sustainability of these effects, investigate additional mediators of happiness, and identify the specific components of the intervention that are most effective. Additionally, extending this study to other contexts, such as addressing hate speech perpetrators, could provide a more comprehensive understanding of how to foster inclusive and supportive school environments. By integrating targeted support for victims with efforts to build classroom cohesion, the HateLess program sets a benchmark for anti‐hate speech initiatives. It contributes to the broader goal of creating resilient and inclusive school communities. Finally, the integration of trauma‐informed educational principles may enhance the effectiveness by providing emotionally safe, empowering, and relationally supportive learning environments, especially for students with prior experiences of marginalization or hate speech.

## Supporting information

HappinessSupp.
