# Supplementary material for: Happiness Through HateLess? Examining the Direct and Indirect Effects of an Anti‐Hate Speech Program on Victimized and Non‐Victimized Youth
Source: J Adolesc. 2025 Jun 1;97(6):1645–55. doi: 10.1002/jad.12525 (PMC12318463; doi:10.1002/jad.12525)
Supplement: Supplementary file 1 — HappinessSupp. [file JAD-97-1645-s001.docx]

**Table S1**

*Correlations and Descriptives Among the Study’s Main Variables*

| **Variable** |  |  |  |  |  |  |
| --- | --- | --- | --- | --- | --- | --- |
| 1. T1 Classroom cohesion | – | .56** | .48** | .36** | -.10* | -.07* |
| 1. T2 Classroom cohesion |  | – | .24** | .49** | -.07* | -.14** |
| 1. T1 Happiness |  |  | – | .47** | -.21** | -.12** |
| 1. T2 Happiness |  |  |  | – | -.22** | -.17** |
| 1. T1 Hate speech victimization |  |  |  |  | – | .47** |
| 1. T2 Hate speech victimization |  |  |  |  |  | – |
| *M* _intervention_ | 2.99 | 3.34 | 2.97 | 3.27 | 1.58 | 1.27 |
| *SD* _intervention_ | 0.82 | 0.62 | 0.81 | 0.75 | 0.99 | 0.58 |
| *M* _control_ | 2.99 | 3.00 | 3.15 | 3.13 | 1.47 | 1.60 |
| *SD* _control_ | 0.82 | 0.82 | 0.85 | 0.89 | 0.84 | 0.93 |

Note: ** *p* < .001 * *p* < .01. T1 = Pretest, T2 = Posttest

| **Table S2**  *Results of the Multilevel Mediation Model in the Total Sample* | | | | |
| --- | --- | --- | --- | --- |
| **Predictor** | **Mediator** | **Outcome** | ***B* (*SE*)** | ***p*** |
| **Direct Effects** |  |  |  |  |
| Group assignment ^intervention^ | Classroom cohesion (T2) |  | .46 (.09) | <.001 |
| Group assignment ^intervention^ |  | Happiness (T2) | .07 (.09) | .438 |
| **Indirect Effects** |  |  |  |  |
| Group assignment ^intervention^ | Classroom cohesion (T2) | Happiness (T2) | .19 (.04) 95% CI [.11, .26] | |
| **Control variables** |  |  |  |  |
| Age |  | Classroom cohesion (T2) | -.06 (.04) | .194 |
| Age |  | Happiness (T2) | -.04 (.02) | .324 |
| Gender ^girls^ |  | Classroom cohesion (T2) | .17 (.07) | .012 |
| Gender ^girls^ |  | Happiness (T2) | .06 (.08) | .470 |
| Immigrant background ^yes^ |  | Classroom cohesion (T2) | .34 (.14) | .013 |
| Immigrant background ^yes^ |  | Happiness (T2) | -.19 (.10) | .072 |
| Classroom cohesion (T1) |  | Classroom cohesion (T2) | .44 (.06) | <.001 |
| Happiness (T1) |  | Happiness (T2) | .37 (.06) | <.001 |
| Note: T1 = Pretest, T2 = Posttest. Reference category: group assignment = control group; gender = boys; immigrant background = no. | | | | |
